# Significant Prediction of In-hospital Major Adverse Events by D-Dimer Level in Patients With Acute Type A Aortic Dissection

**DOI:** 10.3389/fcvm.2022.821928

**Published:** 2022-02-24

**Authors:** Weiqi Feng, Qiuji Wang, Chenxi Li, Jinlin Wu, Juntao Kuang, Jue Yang, Ruixin Fan

**Affiliations:** ^1^School of Medicine, South China University of Technology, Guangzhou, China; ^2^Department of Cardiovascular Surgery, Guangdong Provincial Cardiovascular Institute, Guangdong Provincial People's Hospital, Guangdong Academy of Medical Sciences, Guangzhou, China; ^3^The Second School of Clinical Medicine, Southern Medical University, Guangzhou, China; ^4^Department of Cardiac Surgery Intensive Care Unit, Guangdong Cardiovascular Institute, Guangdong Provincial People's Hospital, Guangdong Academy of Medical Sciences, Guangzhou, China; ^5^Department of Cardiovascular Surgery, Guangdong First People's Hospital, Guangzhou, China

**Keywords:** acute type A aortic dissection, D-dimer, major adverse events, mortality, survival

## Abstract

**Background:**

Acute type A aortic dissection (ATAAD) is a rare, life-threatening condition affecting the aorta. This study explores the relationship between the level of admission D-dimer, which was assessed during the first 2 h from admission, and in-hospital major adverse events (MAE) with ATAAD.

**Methods:**

A total of 470 patients with enhanced computed tomography (CT) confirmed diagnosis of ATAAD who underwent operation treatment in Guangdong Provincial People's hospital between September 2017 and June 2021 were enrolled in the present study. The X-tile program was used to determine the optimal D-dimer thresholds for risk. Restricted cubic spline (RSC) was performed to assess the association between D-dimer and endpoint. The perioperative data were compared between the two groups, univariate and multivariate analyses were used to investigate the risk factors of major adverse events (in-hospital mortality, gastrointestinal bleeding, paraplegia, acute kidney failure, reopen the chest, low cardiac output syndrome, cerebrovascular accident, respiratory insufficiency, MODS, gastrointestinal bleeding, and severe infection).

**Results:**

Among 470 patients, 151 (32.1%) had MAE. In-hospital mortality was 7.44%. The patients with D-dimer >14,500 ng/ml were more likely to present with acute kidney failure, low cardiac output, cerebrovascular accident, multiple organ dysfunction syndromes (MODS), gastrointestinal bleeding, and severe infection. D-dimer level was an independent risk factor for acute kidney failure (OR 2.09, 95% CI: 1.25–3.51, *p* = 0.005), MODS (OR 6.40, 95% CI: 1.23–33.39, *p* = 0.028), gastrointestinal bleeding (OR 17.76, 95% CI: 1.99–158.78, *p* = 0.010) and mortality (OR 3.17, 95% CI: 1.32–7.63, *p* = 0.010). Multivariate regression analysis of adverse events also suggested that D-dimer >14,500 ng/ml (OR 1.68, 95% CI: 1.09–2.61, *p* = 0.020) was the independent risk factor of major adverse events.

**Conclusions:**

Increasing D-dimer levels were independently associated with the in-hospital MAE and thus can be used as a useful prognostic biomarker before the surgery.

## Introduction

According to the Stanford system, dissections involving the ascending aorta are classified as type A, whereas those involving only the descending aorta are classified as type B (1). Acute type A aortic dissection (ATAAD) is a fatal condition with rapid onset and high mortality. The dissection of the aorta allows blood to flow between the layers of the aortic wall, forcing the layers to apart (2). ATAAD accounts for 58–62% of aortic diseases (1). It has been reported that the mortality rates among ATAAD patients who do not receive surgical treatment can reach 30% within 48 h (3). The German aortic dissection study showed that the mortality rate of 2,137 patients within 30 days was 16.9% (4). Although great advances have been made in the level of perioperative care management and surgical technique, there are still many complications, such as death, gastrointestinal bleeding, and paraplegia, which adversely affect the survival of ATAAD patients. Therefore, rapid diagnosis and emergency surgical treatment are crucial for patients.

D-dimer is the breakdown fragment of fibrin and a biomarker of the synthesis of the balance between coagulation and fibrinolysis (5). It is broadly used for the diagnosis of pulmonary embolism and deep vein thrombosis (6). Moreover, previous studies have shown that D-dimer levels are elevated in ATAAD (7, 8). Yet, it remains unclear whether D-dimer could be used as a risk indicator to evaluate in-hospital major adverse events. Consequently, this study was designed to explore the relationship between D-dimer and major adverse events in ATAAD patients.

## Materials and Methods

### Study Setting

Between September 2017 and June 2021, a series of consecutive ATAAD patients underwent emergency surgery. The inclusion criteria for patients in this study were as follows: (1) diagnosis confirmed by CT angiography; (2) ATAAD defined as a dissection that is diagnosed within 14 days after onset of symptoms (9); (3) age >18 years; and (4) D-dimer levels assessed during the first 2 h from admission. Data were collected from the hospital's electronic medical record system. This study has been reviewed by the Ethics Committee of Guangdong Provincial People's Hospital.

### Data Collection

Demographic variables included age, sex, smoking status, drinking status, and body mass index (BMI). Medical history included hypertension, diabetes, bicuspid aortic valve (BAV), Marfan syndrome (MFS), known coronary artery disease (CAD), and previous cardiovascular surgery.

Laboratory exams, performed after admission and before the emergency surgery, included white blood cell count, neutrophil ratio, platelet count, and D-dimer count. D-dimer was measured in our center laboratory by using immunoturbidimetric assay (Diagnostica Stago). The normal upper limit for the D-dimer assay was 500 ng/mL, and the upper detection limit was 20,000 ng/ml.

### Study Endpoints

The major adverse events (MAE) were classified according to the consensus statement from the International Aortic Arch Surgery Study Group (10), including in-hospital mortality, gastrointestinal bleeding, paraplegia, acute kidney failure, reopen the chest, low cardiac output syndrome, cerebrovascular accident, respiratory insufficiency, MODS, gastrointestinal bleeding, and severe infection. Acute kidney failure was defined as serum creatinine increased by >3 times the baseline values, glomerular filtration rate decreased by >75%, or requiring temporary hemodialysis support for the resolution. Low cardiac output syndrome was defined as large doses of vasoactive drugs with signs of hypoperfusion of tissues, requiring intra-aortic balloon pump insertion or requiring extracorporeal membrane oxygenation support. Respiratory insufficiency was defined as pneumonia, atelectasis, acute respiratory distress syndrome, or a tracheostomy.

### Statistical Analyses

The optimal cut-off point for the D-dimer level was determined by the X-tile (version 3.6.1; Rimm Lab; Yale School of Medicine) (11), and find the optimal cut-off thresholds of D-dimer to differentiate patients into high and low risk groups. Continuous variables are presented as the mean ± standard deviation and median (interquartile range) and compared using Student's *t*-test and Mann-Whitney *U*-test. Categorical variables are presented as frequency rates and percentages and compared by χ^2^ tests. Kaplan-Meier method was used to construct the survival curve. Receiver operating characteristics (ROC) curves were constructed for predicting the value of D-dimer, and the area under the curve (AUC) was calculated. Logistic regression models were used to estimate the odds of the study endpoints and identify the independent factors associated with in-hospital MAE. Results of the logistic regression are presented as odds ratios (ORs). Restricted cubic spline (RCS) can assess and visualize the relationship between an independent variable and a dependent variable. We used RCS with fourth knots at the 5th, 35th, 65th, and 95th centiles to flexibly model at relationships of admission D-dimer with in-hospital mortality. Statistical significance was set as a two-sided *P* < 0.05. Stata 17.0 was used for statistical analysis and R software was used for RCS.

## Results

### Patient Characteristics

Among 479 consecutive patients with ATAAD, 9 patients had no D-dimer test and were excluded. All the patients were Asian and most were operated on within 4 days after admission. Patients were divided into two subgroups by using X-tile software: group 1 was composed of patients with preoperative levels of D-dimer ≤ 14,500 ng/ml (*n* = 273, 58.09%), and group 2 was composed of patients with preoperative levels of D-dimer >14,500 ng/ml (*n* = 197,41.91%). The mean age of included patients was 51.86 ± 10.76 years, and 87.26% of the patients were male (Table 1).

**Table 1:** Patient characteristics.

	**All patients**	**D-dimer ≤14,500 ng/ml**	**D-dimer >14,500 ng/ml**	***p*** **-value**
	**(***n*** = 470)**	**(***n*** = 273)**	**(***n*** = 197)**	
**Demographics**				
Age (years)	51.86 ± 10.76	50.39 ± 11.36	54.24 ± 9.52	<0.001
Male gender	396 (84.26%)	227 (83.15%)	169 (85.79%)	0.439
Smoker	161 (34.26%)	98 (35.90%)	63 (31.98%)	0.337
Drinker	35 (7.45%)	17 (6.23%)	18 (9.14%)	0.236
BMI (Kg/m^2^)	24.79 ± 3.94	24.65 ± 3.97	24.75 ± 3.96	0.511
**Medical history**				
Hypertension	321 (68.30%)	153 (64.8%)	168 (71.8%)	0.105
Diabetes	8 (1.7%)	5 (1.83%)	3 (1.52%)	0.799
History of cardiovascular surgery	37 (7.87%)	34 (12.45%)	3 (1.52%)	<0.001
CAD	46 (9.79%)	25 (9.15%)	21 (10.66%)	0.589
MFS	24 (5.11%)	20 (7.33%)	4 (2.03%)	0.010
BAV	10 (2.16%)	9 (3.30%)	1 (0.51%)	0.039
Aspirin	11 (2.34%)	7 (2.56%)	4 (2.03%)	0.706
**Admission laboratory results**				
White blood cell count (×10^9^)	12.48 (10.22–15.20)	11.52 (9.43–13.97)	13.77 (11.61–16.38)	<0.001
Neutrophil ratio	0.822 (0.757–0.861)	0.785 (0.723–0.835)	0.849 (0.808–0.886)	<0.001
Platelets (×10^9^)	185 (151–228)	202 (164–253)	164 (131–200)	<0.001
D-dimer (ng/ml)	10,120 (3,450–20,000)	4,160 (1,590–8,070)	20,000 (20,000–20,000)	<0.001
**Echocardiographic**				
LVEF	63.80 ± 6.41	63.43 ± 6.55	64.31 ± 6.19	0.147
AAO	43.64 ± 7.22	44.09 ± 7.95	43.02 ± 6.02	0.116
LA	34.12 ± 4.95	34.39 ± 4.90	33.73 ± 5.00	0.160
LVESD	30.23 ± 5.96	31.40 ± 2.25	29.01 ± 5.58	<0.001
**AR**				0.160
None/mild	313 (66.60%)	181 (66.30%)	132 (67.01%)	
Moderate	88 (18.72%)	47 (17.22%)	41 (20.81%)	
Severe	61 (12.98%)	40 (14.65%)	21 (10.66%)	
**MR**				0.111
None/mild	442 (97.04%)	256 (93.77%)	189 (95.94%)	
Moderate	13 (2.77%)	10 (3.66%)	3 (1.52%)	
Severe	3 (0.64%)	2 (0.73%)	1 (0.51%)	
**TR**				0.855
None/mild	435 (92.5%)	251 (91.94%)	184 (93.40%)	
Moderate	22 (4.68%)	14 (5.13%)	8 (4.06%)	
Severe	4 (0.85%)	3 (1.10%)	1 (0.51%)	
**Procedural information**				
CPB time	241.0 (211.0–280.0)	234.0 (202.0–265.0)	258.0 (217.5–296.5)	<0.001
ACC time	132.0 (103.0–160.0)	132.0 (103.0–160.0)	127.0 (101.0–151.0)	<0.001
CABG	32 (6.81%)	12 (4.40%)	20 (10.15%)	0.014
Total arch replacement	452 (96.17%)	256 (93.77%)	196 (99.49%)	0.001

*Smoker is defined as current smoker (smoke more than 100 cigarettes and has smoked in the last 1 month) and ex-smoker*.

*BMI, body mass index; BAV, bicuspid aortic valve; MFS, Marfan syndrome; CAD, coronary artery disease; LVEF, left ventricular ejection fraction; AAO, ascending arota; LA, left atrium; LVESD, left ventricular end diastolic diameter; AR, aorta ascendens; MR, mitral regurgitation; TR, tricuspid regurgitation; CPB, cardiopulmonary bypass; ACC, aortic cross clamp; CABG, coronary artery bypass graft*.

Compared with group1 patients (D-dimer ≤ 14,500 ng/ml), group 2 patients (D-dimer >14,500 ng/ml) were older (*p* < 0.001), and their preoperative left ventricular end diastolic diameter were larger (*p* < 0.001). Group2 patients had higher levels of preoperative white blood cell count (*p* < 0.001) and platelet count (*p* < 0.001). Group2 patients had longer cardiopulmonary bypass time (*p* < 0.001). In terms of coronary artery bypass graft (*p* = 0.014), and total arch replacement (*p* = 0.001) were also more commonly found in group2 patients. However, the history of cardiovascular surgery (*p* < 0.001), BAV (*p* = 0.039), and MFS (*p* = 0.010) were more commonly found in patients with D-dimer ≤ 10,120 ng/ml. There were no statistically significant differences in other variables (Table 1).

### D-Dimer and All Adverse Events

In all patients, 89 (18.94%) patients had acute kidney failure; 35 (7.44%) patients died; 28 (5.96%) patients had respiratory insufficiency; 27 (5.74%) patients had the cerebrovascular accident; 25 (5.32%) patients had low cardiac output syndrome; 19 (4.04%) patients had paraplegia; 15 (3.19%) patients had severe infection; 12 (2.55%) patients had MODS; 11 (2.34%) patients had gastrointestinal bleeding; and 7 (1.49%) patients needed to reopen the chest.

There were no significant differences between the two groups in paraplegia, reopen the chest and respiratory insufficiency. Group2 patients were more likely to present with acute kidney failure (27.92 vs.12.45%, *p* < 0.001), low cardiac output (8.63 vs. 2.93%, *p* = 0.007), cerebrovascular accident (8.63 vs. 2.93%, *p* = 0.007), multiple organ dysfunction syndrome (MODS) (5.08 vs. 0.73%, *p* = 0.003), gastrointestinal bleeding (5.08 vs. 0.37%, *p* = 0.001), and severe infection (5.58 vs.1.47%, *p* < 0.001) (Table 2). Unadjusted analysis showed that D-dimer level >14,500 ng/ml was associated with increased mortality and incidences of MAE. After adjustment, D-dimer level was an independent risk factor for acute kidney failure (OR 2.09, 95% CI: 1.25–3.51, *p* = 0.005), MODS (OR 6.40, 95% CI: 1.23–33.39, *p* = 0.028), gastrointestinal bleeding (OR 17.76, 95% CI: 1.99–158.78, *p* = 0.010) and mortality (OR 3.17, 95% CI: 1.32–7.63, *p* = 0.010) (Table 3).

**Table 2:** In-hospital MAE patients with ATAAD.

	**All patients**	**D-dimer ≤14,500 ng/ml**	**D-dimer >14,500 ng/ml**	***p*** **-value**
	**(***n*** = 470)**	**(***n*** = 273)**	**(***n*** = 197)**	
Major adverse events	151 (32.13%)	66 (24.18%)	85 (43.15%)	<0.001
Acute kidney failure	89 (18.94%)	34 (12.45%)	55 (27.92%)	<0.001
Death	35 (7.44%)	9 (3.30%)	26 (13.20%)	<0.001
Cerebrovascular accident	27 (5.74%)	9 (3.30%)	18 (9.14%)	0.007
Respiratory insufficiency	28 (5.96%)	13 (4.76%)	15 (7.61%)	0.197
Low cardiac output syndrome	25 (5.32%)	8 (2.93%)	17 (8.63%)	0.007
paraplegia	19 (4.04%)	9 (3.30%)	10 (5.08%)	0.334
Severe infection	15 (3.19%)	4 (1.47%)	11 (5.58%)	0.001
MODS	12 (2.55%)	2 (0.73%)	10 (5.08%)	0.003
Gastrointestinal bleeding	11 (2.34%)	1 (0.37%)	10 (5.08%)	0.001
Reopen the chest	7 (1.49%)	5 (1.83%)	2 (1.02%)	0.471

**Table 3:** Odds radio by D-dimer levels for MAE.

	**Unadjusted OR**		**Adjusted OR**	
	**OR (95% CI)**	***p*** **-value**	**OR (95% CI)**	***p*** **-value**
Gastrointestinal bleeding	14.55 (1.85–114.59)	0.011	17.76 (1.99–158.78)	0.010
MODS	7.25 (1.57–33.45)	0.011	6.40 (1.23–33.39)	0.028
Death	4.46 (2.04–9.75)	<0.001	3.17 (1.32–7.63)	0.010
Severe infection	3.98 (1.25–12.68)	0.020	2.68 (0.78–9.17)	0.117
Acute kidney failure	2.72 (1.69–4.38)	<0.001	2.09 (1.25–3.51)	0.005
Cerebrovascular accident	2.95 (1.30–6.71)	0.010	1.90 (0.79–4.60)	0.153
Low cardiac output syndrome	3.13 (1.32–7.40)	0.009	1.80 (0.68–4.78)	0.238

*Adjusted model for age, hypertension, white blood cell count, D-dimer levels, CPB time, ACC time, and CABG*.

### D-Dimer and Mortality

**Figure 1:**
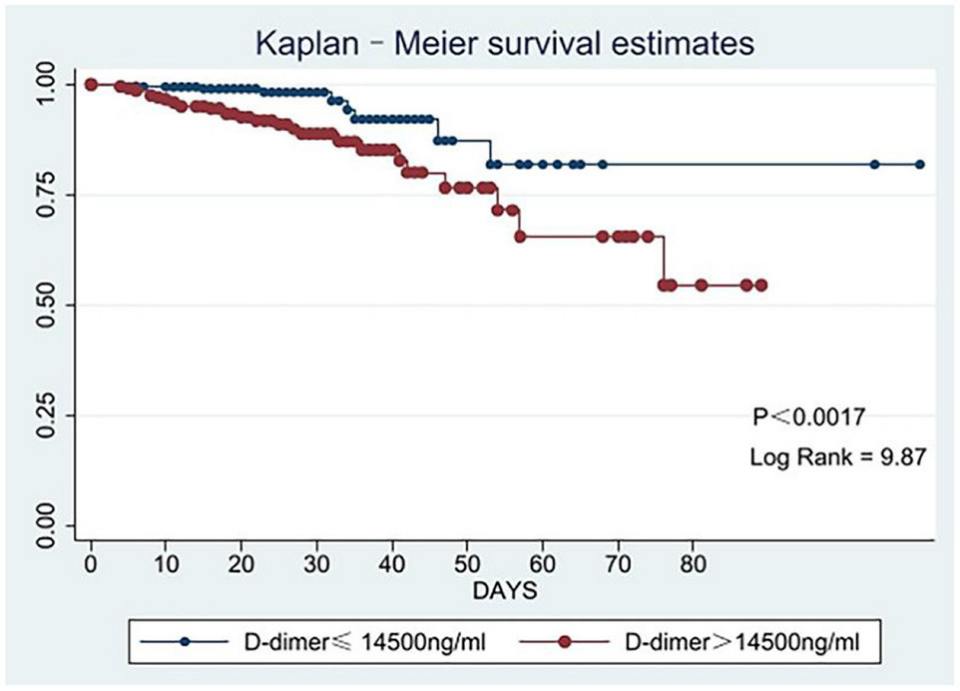
Kaplan-Meier survival curve analyses for the in-hospital mortality according to low and high levels of D-dimer.

**Figure 2:**
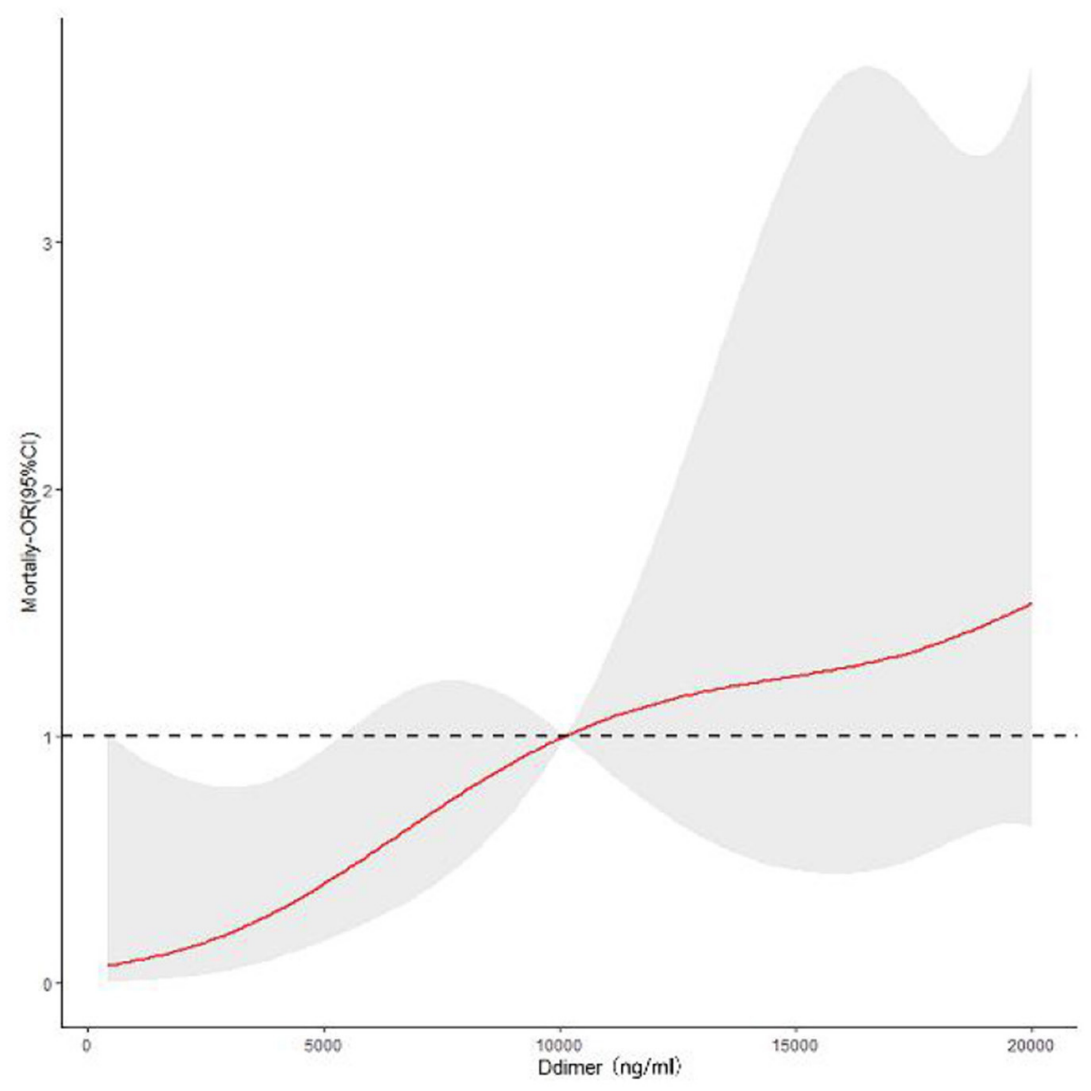
Continuous odds ratio across D-dimer levels for mortality according to restricted cubic spline.

The in-hospital mortality rate was 7.44%. As shown in Table 2, the group2 patients had a higher in-hospital mortality rate (13.20 vs. 3.30%, *p* < 0.001). Our finds showed an independent association between D-dimer levels and mortality (OR 3.17, 95% CI: 1.32–7.63, *p* = 0.010). As shown in Figure 1, there were significant differences in the cumulative probability of the overall survival between the two groups. The RCS analysis showed that the risk of in-hospital mortality was flat until 10,000 ng/ml of D-dimer and then started to increase quickly (Figure 2).

### Risk Factors for MAE

151 (32.13%) patients had in-hospital MAE. Group2 patients were more likely to have in-hospital MAE (43.15 vs. 24.18%, *p* < 0.001) than group1 patients. Univariate and multivariate analyses for MAE were presented in Table 4. Univariate logistic regression showed that age, hypertension, white blood cell count, the D-dimer levels >14,500 ng/ml, CPB time, ACC time, and CABG were the risk indicators for MAE. Multivariate logistic regression model, which included age, hypertension, white blood cell count, D-dimer, CPB time, ACC time, and CABG, was then created and showed that the D-dimer > 14,500 ng/ml (OR 1.68, 95% CI 1.09–2.61, *p* = 0.020), hypertension (OR 1.75, 95% CI 1.08–2.83, *p* = 0.022) and CPB time (OR 1.01, 95% CI 1.00–1.01, *p* = 0.045) were significantly associated with a higher risk of MAE.

**Table 4:** Univariate and multivariate analysis for MAE in patients with ATAAD.

	**Univariate analysis**			**Multivariate analysis**		
**Variable**	**OR**	**95% CI**	***p*** **-value**	**OR**	**95% CI**	***p*** **-value**
Age	1.03	1.01–1.05	0.003	1.02	1.00–1.04	0.072
Male/Gender	0.98	0.58–1.67	0.951			
Hypertension	1.94	1.24–3.03	0.004	1.75	1.08–2.83	0.022
BMI	1.02	0.97–1.07	0.424			
CAD	1.41	0.75–2.63	0.286			
BAV	0.52	0.11–2.47	0.410			
Diabetes	2.14	0.53–8.69	0.286			
Aspirin	0.45	0.10–2.12	0.315			
White blood cell count (×10^9^)	1.08	1.02–1.14	0.004	1.05	0.99–1.12	0.064
D-dimer >14,500 ng/ml	2.38	1.60–3.53	<0.001	1.68	1.09–2.61	0.020
LVESD	0.98	0.95–1.01	0.215			
CPB time	1.01	1.00–1.01	<0.001	1.01	1.00–1.01	0.045
CABG	4.52	2.12–9.65	<0.001	2.33	0.95–5.70	0.064
ACC	1.01	1.00–1.02	<0.001	1.00	0.99–1.01	0.802
Total arch replacement	1.24	0.43–3.55	0.687			

*BMI, body mass index; CAD, coronary artery disease; CPB, cardiopulmonary bypass; CABG, coronary artery bypass graft; ACC, aortic cross clamp*.

**Figure 3:**
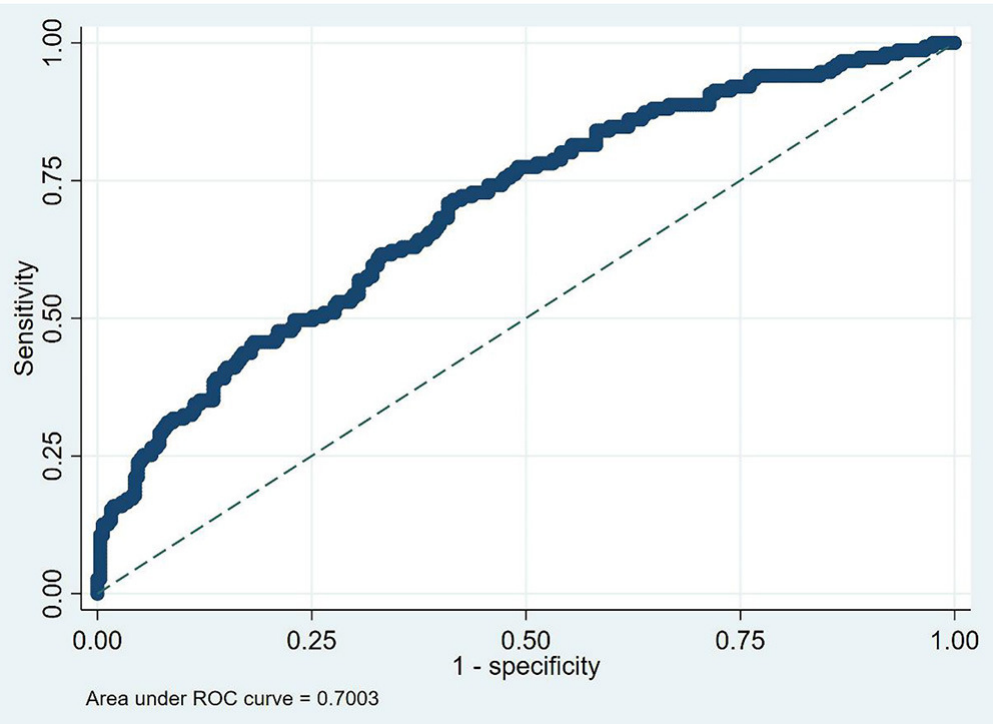
Predict for MAE.

**Figure 4:**
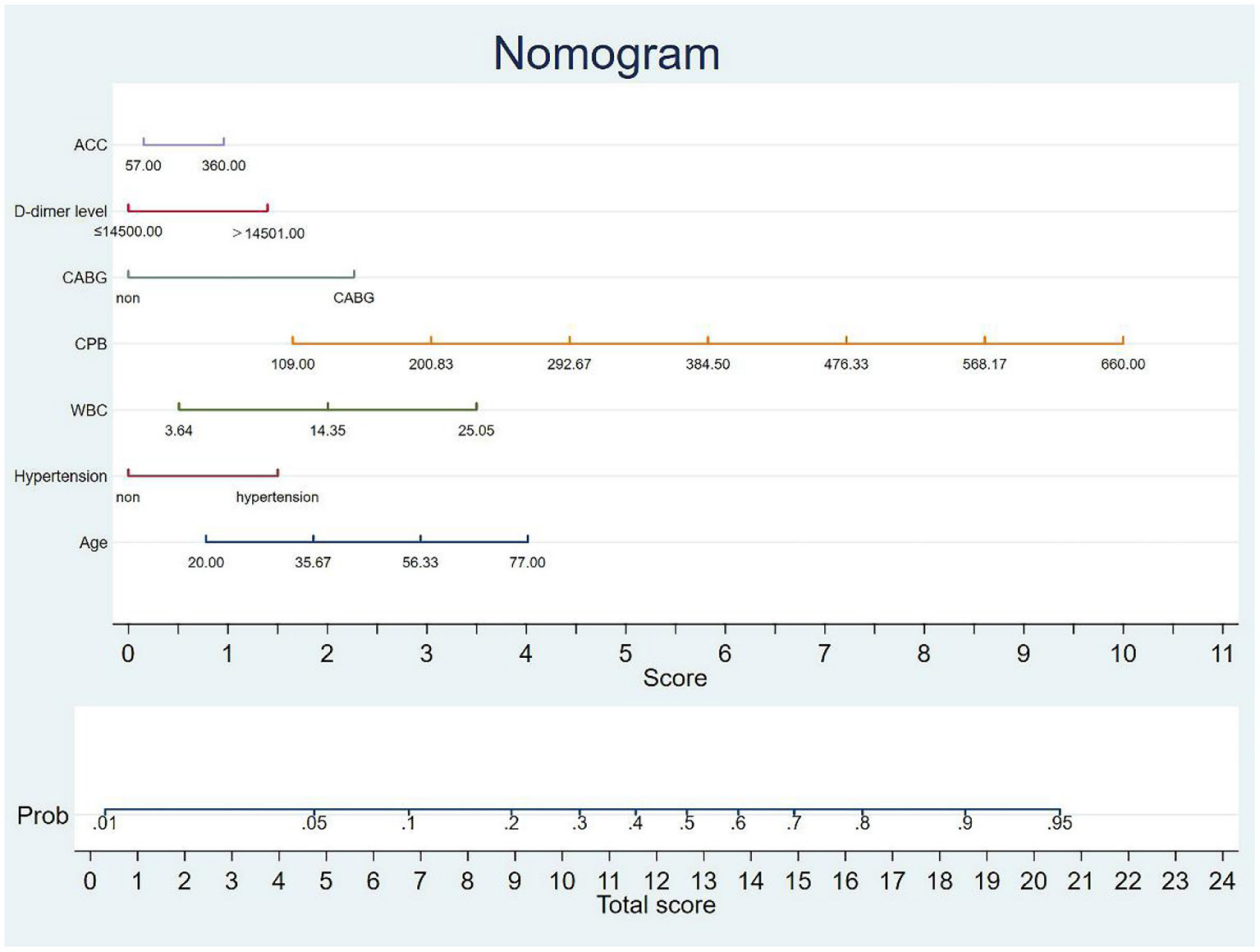
Nomogram for MAE.

The ROC curve for the model was illustrated in Figure 3 (AUC = 0.7003, *p* < 0.001). And the nomogram for the model to assess the risk of MAE was illustrated in Figure 4.

## Discussion

The D-dimer is very sensitive to intravascular thrombus and may be markedly elevated in disseminated intravascular coagulation, acute aortic dissection, and pulmonary embolism (12). In the past years, many studies have shown that there is a certain relationship between D-dimer and AD. The IRAD-Bio study (13) identified D-dimer should suggest being useful as a rule-out diagnostic tool and individualize patients within 24 h after onset. Ohlmann et al. (8) found patients with acute chest pain and elevated D-dimer level should consider a diagnosis of AD. Fan et al. (14) revealed that D-dimer could be used as a potential biomarker for suspected patients with ATAAD. Therefore, the D-dimer test is more and more widely used in the diagnosis of ATAAD despite not being very specific.

Our study has shown that D-dimer 14,500 ng/ml was an independent risk factor for major adverse events. The X-tile, which can present a tool for assessment of relationships between a biomarker and outcome and the discovery of population cut-points based on biomarker expression, helps us to effectively differentiate the levels of D-dimer into low and high risks, which could have the potential to prompt for more tailored risk treatment. Our findings improve the understanding of major adverse events in ATAAD, increase clinical vigilance, help to assist clinical decision and treat strategy.

Different countries have different lifestyles and most international research included patients from developed countries with better health systems that could not reflect the real condition in China. The age of the patients (51.86 ± 10.76) in our study was younger compared with the age of patients (60.6 ± 14.8) in the International Registry of Acute Aortic Dissection Substudy on Biomarkers (IRAD-Bio study) (13) and the patients (60.5 ± 13.6) in the German Registry for Acute Aortic Dissection Type A (GERAADA) (4). More young people had an aortic dissection in China than in Western countries that need to pay attention to. Compared with results from other studies, more patients were male (84.26%) in our study. One of the reasons may be that the prevalence of tobacco use in Chinese men is one of the highest in the world (15). Smoking increases the risk of cardiovascular disease, and smoking-related mortality is increasing in China. Compared with the smoking rate in the general Chinese population (33.5%) (16), the smoking rate was similar (34.26%) in our study.

The IRAD-Bio study (13) found that patients in type B aortic dissection (3,213 ± 1,465 ng/ml) had lower levels of D-dimer compared with patients in type A aortic dissection (3,574 ± 1,430 ng/ml), which indicated that D-dimer would be affected by different types of dissection.

In our study, the in-hospital mortality rate was 7.44%; therefore, the emergence of surgical treatment is of vital importance for saving people's lives. For patients with D-dimer ≤14,500 ng/ml, the in-hospital mortality was 3.30%; for patients with D-dimer > 14,500 ng/ml, the in-hospital mortality was 13.20%. Patients with D-dimer >14,500 ng/ml had a higher risk of death. The survival curve and RCS clearly showed that the in-hospital mortality rate with elevated D-dimer levels was higher. Previous studies found a correlation between the risk of death and D-dimer levels, which was further confirmed in our study. Ohlmann et al. (8) found that D-dimer >5,200 ng/ml is related to mortality, while Weber et al. (7) found that D-dimer can be an independent indicator of mortality in ATAAD patients. Therefore, there was a positive link between D-dimer levels and in-hospital mortality rate.

In addition, our finds also indicated that elevated D-dimer levels were related to acute kidney failure, severe infection, and gastrointestinal bleeding. The elevated D-dimer patients had a significant relationship with acute kidney failure. Acute kidney failure is a common complication following ATAAD surgery. Patients who developed acute kidney failure were more likely to present with a dissection involving the ascending and descending aorta and more commonly presented with cardiac tamponade (17). Gastrointestinal bleeding is an uncommon complication in ATAAD, leading to a high mortality rate (18). In our study, 12 patients suffered from gastrointestinal bleeding, and eight patients died. D-dimer levels were positively associated with gastrointestinal blooding. Accordingly, patients should undergo conventional stool examination after surgery to early discovery and significantly reduce their mortality rate.

Logistic regression also showed that CPB duration and hypertension were associated with MAE. Some previous studies (19–21) have illustrated that longer CPB time was associated with a greater possibility of suffering from MAE (OR = 1.01), which was similar to our results. Hypertension was not only one of the most important risk factors for ATAAD (1, 22), but it also resulted as the main risk factor for MAE in the present study. High blood pressure would damage blood vessels and do harm to our health. Therefore, good control of blood pressure is extremely important for health. In addition, there were significant differences in the known history of cardiovascular surgery that were more common among patients with decreased D-dimer. The reason is unclear. Maybe the patients with a known history of cardiovascular surgery in group1 received more follow-up over time and therefore may take less time to arrive at the hospitalization than the patients in group2. More patients with a known history of cardiovascular surgery have taken antithrombotic therapy in the past. Antiplatelet can inhibit the adhesion and aggregation function of platelet, thus producing antithrombotic effects. Anticoagulant can inhibit thrombin so that they inhibit fibrinogen from transforming into fibrin. As we all know, D-dimer is a degradation product of fibrin (5). Therefore, antithrombotic therapy leads to the decrease of D-dimer levels. The D-dimer level reflects the disordered condition of coagulation.

The study has some limitations. First, it is a retrospective observational study that enrolled patients in a single center. Consequently, there might be some admission bias, and future prospective studies are needed to confirm the role of D-dimer further. Next, the study only focused on the admission D-dimer levels, whether postoperative D-dimer is a meaningful marker that needs further investigation. And the data about anticoagulants is not available in our research. Greater sample size, more D-dimer subgroups, more complete data, and longer follow-up time are needed to further verify reported findings.

## Conclusion

Early evaluation of d- dimer after admission seems to represent a useful, rapid, and cheap prognostic biomarker, which can individualize ATAAD patients with high mortality and MAE risk. D-dimer may be used as a complementary tool for the diagnostic, that can help decision and treatment making.

## Data Availability Statement

The raw data supporting the conclusions of this article will be made available by the authors, without undue reservation.

## Author Contributions

WF: analyzing data and writing the manuscript. QW: writing the manuscript. JK and JY: acquisition of data. CL and JW: revising it critically for important content. RF: editing and approving of the version. All authors contributed to the article and approved the submitted version.

## Conflict of Interest

The authors declare that the research was conducted in the absence of any commercial or financial relationships that could be construed as a potential conflict of interest.

## Publisher's Note

All claims expressed in this article are solely those of the authors and do not necessarily represent those of their affiliated organizations, or those of the publisher, the editors and the reviewers. Any product that may be evaluated in this article, or claim that may be made by its manufacturer, is not guaranteed or endorsed by the publisher.
